# A Lepromatous Leprosy Patient with Permanent Disability

**DOI:** 10.4269/ajtmh.14-0843

**Published:** 2015-11-04

**Authors:** Na Wang, Hong Liu, Furen Zhang

**Affiliations:** Shandong Provincial Hospital for Skin Diseases, Shandong University, Shandong, People's Republic of China; Shandong Provincial Institute of Dermatology and Venereology, Shandong Academy of Medical Sciences, Shandong, People's Republic of China; Shandong Provincial Key Lab for Dermatovenereology, Shandong, People's Republic of China; Shandong Provincial Medical Center for Dermatovenereology, Shandong, People's Republic of China

**Figure 1.**
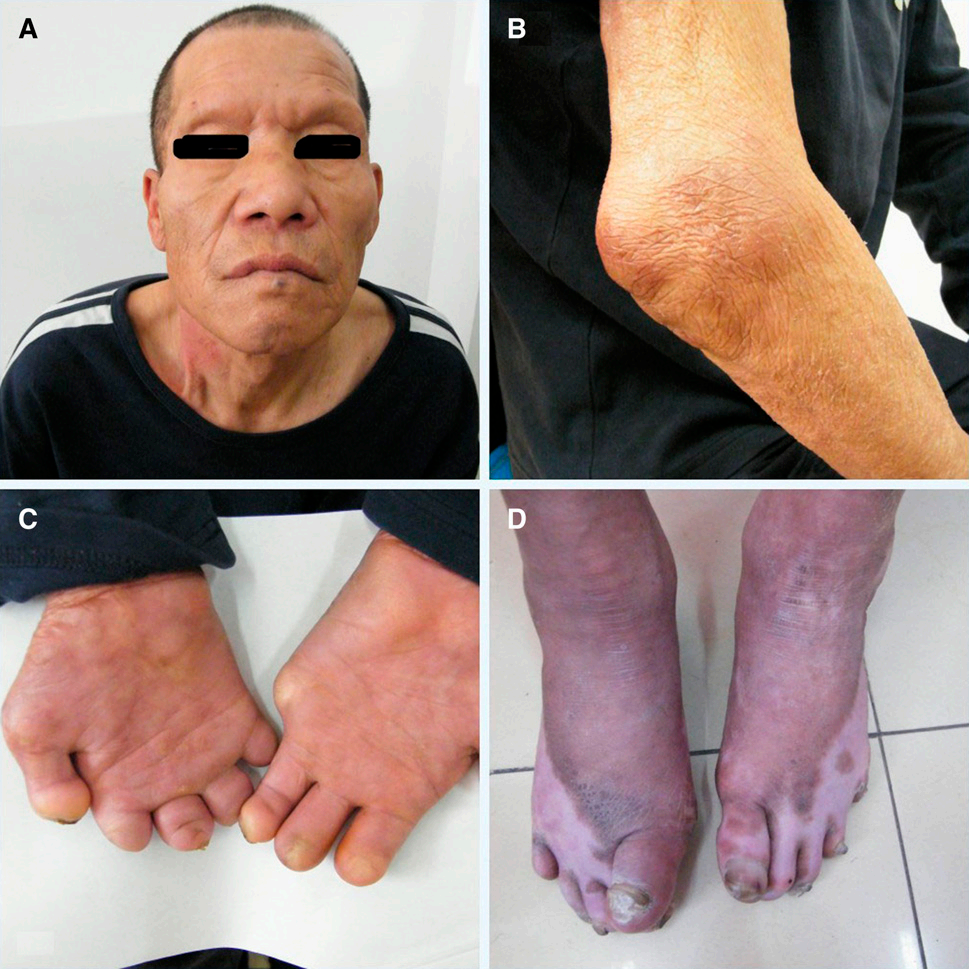
Clinical features of the patient showing (**A**) milphosis, (**B**) multiple asymptomatic nonsymmetrical infiltrated nodules on his arm, and both (**C**) hands and (**D**) feet with visible deformities.

**Figure 2.**
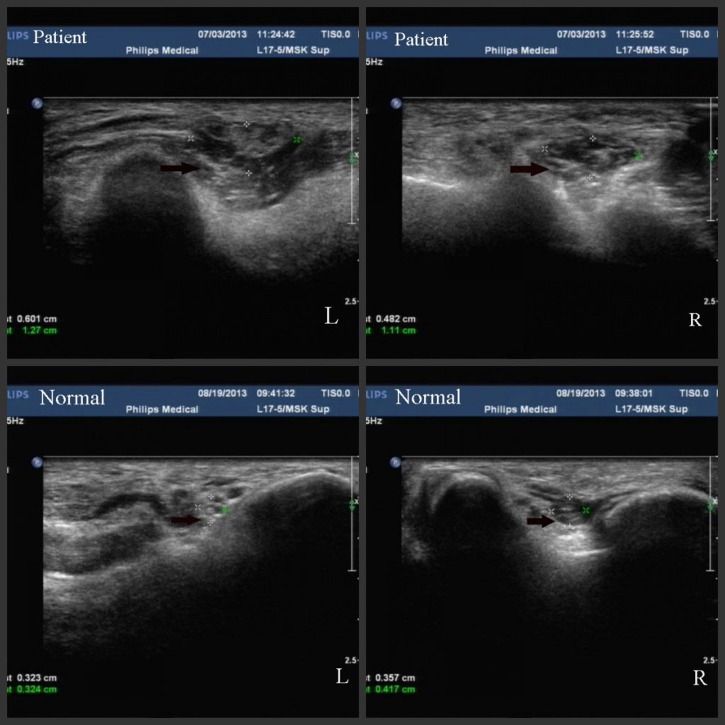
High-resolution sonography of ulnar nerve examination demonstrated diffused swelling of the ulnar nerve at the elbow. Horizontal scan of the left and right side ulnar nerve with hypoechoic fascicles (patient L and R ulnar nerves), while the normal one illustrates the screen mesh structure and the normal size.

A disabled, 61-year-old Chinese man was referred to our hospital with suspected leprosy. Multiple, asymptomatic, and asymmetrical infiltrated nodules were found on his extremities, with loss of eyebrows (Figure 1A and B

). Both hands and feet had developed visible deformities (Figure 1C and D). High-resolution sonography, used for superficial nerve assessment (Figure 2

), showed ulnar nerve trunk inflammation. After being diagnosed with leprosy 18 years ago, because of the stigma of leprosy, he rejected the diagnosis and refused to take medicine. He was subsequently lost in follow-up. During the past 3 years, he gradually developed visible deformities.

**Figure 3.**
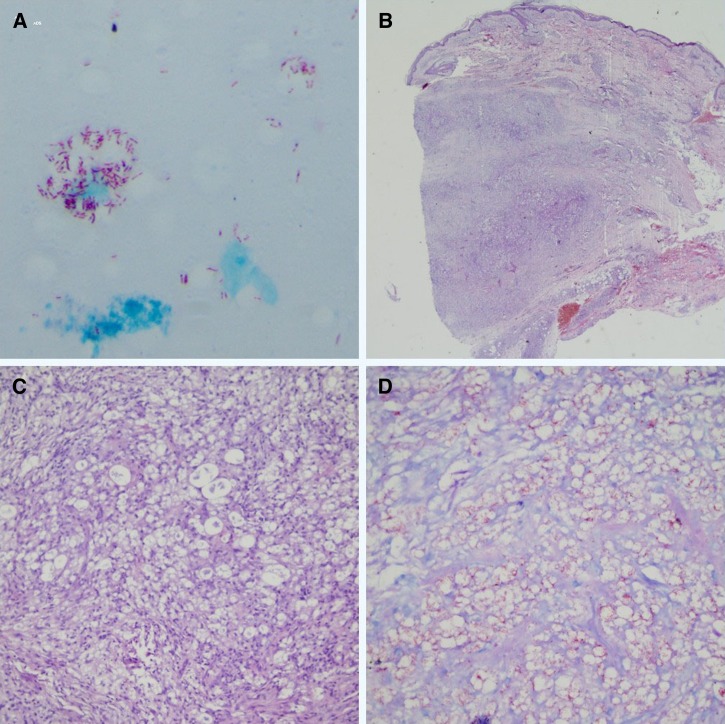
Results of the slit-skin smear and skin biopsy. (**A**) Globus leprosus, (**B**, **C**) skin biopsies showing diffuse dermal infiltrate of foamy histiocytes and numerous bacilli (5+) in the histopathological examinations (**B**: HE ×100; **C**: HE ×400), and (**D**) positive staining of lepra bacilli (6+) (acid-fast bacilli [AFB] stain).

A slit-skin smear test and biopsy confirmed an acid-fast bacilli infection (Figure 3A–D

). A differential polymerase chain reaction test was conducted as previously described,^1^ revealing *Mycobacterium leprae* and confirming the diagnosis of lepromatous leprosy. The patient was treated with rifampicin, dapsone, and clofazimine (multidrug regimen).

Society has stigmatized people infected with leprosy since ancient times, and, once diagnosed, their economic situation may decline, their marital partners may reject them, and opportunities for further education may be reduced.^2^ Several aspects of this case might be instructive of the epidemiological and clinical features of leprosy caused by *M*. *leprae*. Skin nodules usually appear in long-standing leprosy,^3^ just as in our patient. He had had a long history of leprosy, and his lack of awareness led to a major delay in the treatment and subsequent deformity and disability, all of which reminds us that popularizing the knowledge of leprosy is as important as early diagnosis and treatment.
